# Wnt expression and canonical Wnt signaling in human bone marrow B lymphopoiesis

**DOI:** 10.1186/1471-2172-7-13

**Published:** 2006-06-29

**Authors:** Guri Døsen, Ellen Tenstad, Marit Kveine Nygren, Heidi Stubberud, Steinar Funderud, Edith Rian

**Affiliations:** 1 Department of Immunology, Institute for Cancer Research, Rikshospitalet-Radiumhospitalet Medical Center, Medical Faculty, University of Oslo, Norway

## Abstract

**Background:**

The early B lymphopoiesis in mammals is regulated through close interactions with stromal cells and components of the intracellular matrix in the bone marrow (BM) microenvironment. Although B lymphopoiesis has been studied for decades, the factors that are implicated in this process, both autocrine and paracrine, are inadequately explored. Wnt signaling is known to be involved in embryonic development and growth regulation of tissues and cancer. Wnt molecules are produced in the BM, and we here ask whether canonical Wnt signaling has a role in regulating human BM B lymphopoiesis.

**Results:**

Examination of the mRNA expression pattern of Wnt ligands, Fzd receptors and Wnt antagonists revealed that BM B progenitor cells and stromal cells express a set of ligands and receptors available for induction of Wnt signaling as well as antagonists for fine tuning of this signaling. Furthermore, different B progenitor maturation stages showed differential expression of Wnt receptors and co-receptors, β-catenin, plakoglobin, LEF-1 and TCF-4 mRNAs, suggesting canonical Wnt signaling as a regulator of early B lymphopoiesis. Exogenous Wnt3A induced stabilization and nuclear accumulation of β-catenin in primary lineage restricted B progenitor cells. Also, Wnt3A inhibited B lymphopoiesis of CD133^+^CD10^- ^hematopoietic progenitor cells and CD10^+ ^B progenitor cells in coculture assays using a supportive layer of stromal cells. This effect was blocked by the Wnt antagonists sFRP1 or Dkk1. Examination of early events in the coculture showed that Wnt3A inhibits cell division of B progenitor cells.

**Conclusion:**

These results indicate that canonical Wnt signaling is involved in human BM B lymphopoiesis where it acts as a negative regulator of cell proliferation in a direct or stroma dependent manner.

## Background

In mammals, the early antigen independent phase of B lymphopoiesis takes place in the intersinusoidal spaces in the bone marrow (BM). Here, the B cell progeny mature from hematopoietic stem cells (HSC) via early lymphoid progenitors (ELP, comprising common lymphoid progenitors and early B), pro-B, pre-B and immature B developmental stages characterized by successive steps in the rearrangement of immunoglobulin genes and consecutive expression of cellular markers [1-3]. Using immunohistochemical doublestaining we have revealed earlier that all developmental stages of the B cell lineage in human BM tissue are in close contact with slender CD10^+ ^stromal cells or their extensions [4]. This finding correlates with the consensus that B lymphopoiesis is tightly regulated by signals provided by mesenchymal stromal cells and components of the intracellular matrix in the BM microenvironment *in vivo *[4-6]. However, the elements of this signaling are yet inadequately identified; stromal factors like IL 7, Flt3 ligand [7], IL-3 [8,9] and SDF1 [10,11] are essential, but not sufficient for BM B lymphopoiesis [2]. Clearly, there is a need for further characterization of both the stromal phenotype as well as the autocrine and paracrine factors that participate in the regulation of BM B lympopoiesis.

Wnt proteins belong to a large and highly conserved family of secreted, cystein-rich glycoprotein signaling molecules, consisting of 19 members. They are likely to act locally because of their limited solubility [12] and tendency to associate with the cell surface extracellular matrix [13]. Signaling is initiated by Wnt proteins binding to receptors of the Frizzled family (Fzd) on the cell surface. This binding is promiscuous and the ligand/receptor specificities are not yet properly determined. Depending on particular Wnt/Fzd combinations, at least three signaling cascades may be activated. Most studied is the canonical Wnt pathway, which is activated by members of the Wnt1 class (such as Wnt1, Wnt2, Wnt3 and Wnt8) [14]. A key regulatory molecule in this pathway is β-catenin, which in the absence of a Wnt signal is kept low through continuous phosporylation by glycogen synthase kinase-3β (GSK-3β), resulting in a subsequent proteasome dependent destruction of β-catenin. Binding of Wnt ligands to Fzd receptors and coreceptors LRP5/6, leads to inactivation of GSK3β and thereby accumulation of nonphosphorylated β-catenin, which enter the nucleus. Here, β-catenin acts as a coactivator of members of the lymphoid enhancer factor-1 (LEF-1)/T-cell factor (TCF) family of transcription factors to stimulate transcription of Wnt target genes [15]. Activation of Wnt signaling can be inhibited by soluble antagonists, including the Dickkopf (Dkk) family and the soluble Fzd related proteins (sFRP) [16].

Recently, Wnt proteins have drawn attention as a set of factors operating in embryonic development, growth regulation of adult tissues and cancer formation [15,17-20]. Moreover, Wnt signaling plays a central role in the communication between HSC and stromal cells [21] as well as in several other stem cell niches [22,23]. Several observations have established direct roles for Wnt signaling in the maturation process where hematopoietic stem cells lose their pluripotency and commit to specific lineages [24-26]. LEF-1 and Fzd9 knockout mice show defect B lymphopoiesis [24,27] and Wnt signaling seems to be involved in development of leukemia [28-30] and malignant myeloma [31]. Moreover, in murine B lymphopoiesis this signaling pathway has a stimulatory effect on pro-B cells from fetal liver [24]. As early B lymphopoiesis in mice and humans to a certain extent shows distinct factor dependency [32], and since fetal and adult lymphopoiesis takes place in different maturation niches, the aim of the present study was to investigate Wnt signaling in human BM B lymphopoiesis in more detail. We have examined which Wnt signaling pathway molecules that are expressed in B progenitor cells and stromal cells from human BM, and analyzed the regulated expression of several Wnt receptors (Fzd and LRP), β-catenin and plakoglobin as well as the central transcription factors LEF-1 and TCF-4 during the early B lymphopoiesis. Furthermore, we have investigated the effect of recombinant Wnt3A on progenitor B cells. We found that Wnt3A induced β-catenin stabilization and inhibited *in vitro *B lymphopoiesis in a coculture with stromal cells by suppression of initial cell proliferation. Thus, canonical Wnt signaling may be involved in human BM B lymphopoiesis.

## Results

### A distinct set of Wnt ligands, Fzd receptors and Wnt antagonists is expressed in B progenitor cells and stromal cells from human BM

**Figure 1:**
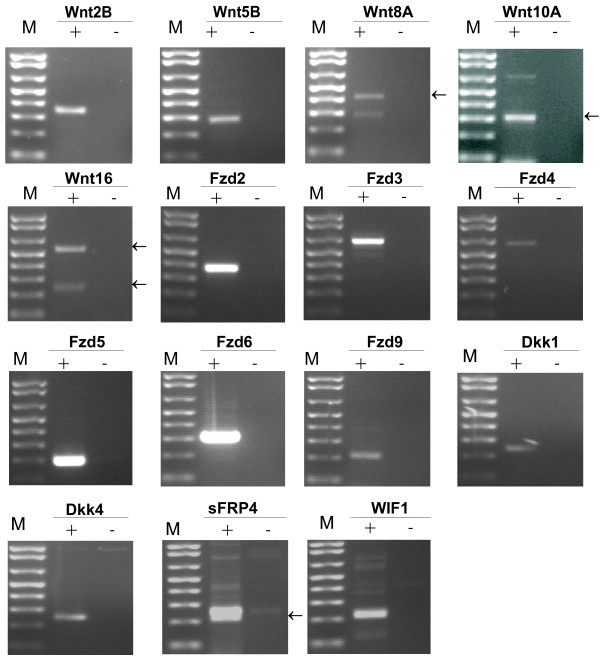
**mRNA expression analyses of Wnt ligands, Fzd receptors and Wnt antagonists**. RT-PCR detection of mRNAs for Wnt ligands, Fzd receptors and Wnt antagonists in BM B progenitor cells. The + and - symbols indicate the presence and absence of reverse transcriptase in the reaction mix, respectively. One representative of two experiments is shown. Amplicon sizes: Wnt2B: 328 bp, Wnt5B:279 bp, Wnt8A: 400 bp, Wnt10A: 296 bp, Wnt16: 520 bp, Fzd2: 306 bp, Fzd3: 622 bp, Fzd4: 605 bp, Fzd5: 197 bp, Fzd6: 300 bp, Fzd9: 210 bp, sFRP4: 243 bp, WIF1: 200 bp, Dkk1: 235 bp, Dkk4: 241 bp. M: Size marker 1 kb Plus DNA ladder (Invitrogen, USA). Where two different bands are detected, an arrow marks the correct band.

Previous work has demonstrated expression of Wnt5A, Wnt2B and Wnt10B in pooled human BM populations [26]. However, the expression pattern of Wnt ligands, Fzd receptors and Wnt antagonists in human B lineage cells has not been explored. In the absence of available antibodies to detect these large families of proteins, we performed conventional RT-PCR on RNA isolated from FACS sorted B progenitor cells (CD10^+^IgM^-^CD45^+^) pooled from three different donors, using primers designed specifically to detect mRNA expression of all known Wnt ligands and Fzd receptors as well as the Wnt antagonists Dkk1, Dkk4, sFRP1-4 and WIF1 (fig. 1 and table 1). In B progenitor cells, Wnt 2B, 5B, 8A, 10A and 16 mRNAs were readily detected. Interestingly, the Wnt16 PCR product had two bands of 520 bp and 233 bp, respectively (fig. 1). The 520 bp band represents the full-length form and the 233 bp band represents a possible splice variant lacking exon 3, potentially giving rise to a truncated Wnt16 form. In addition, expression of several other Wnt mRNAs was detectable, however, less readily (table 1). The Fzd receptors showed on average much higher mRNA expression levels than the Wnts, where Fzd2, 3, 4, 5, 6 and 9 mRNAs were easily detectable in the B progenitor population, as demonstrated by strong PCR bands. Fzd1 and Fzd7 mRNA expression was also demonstrated, but at lower levels than the other Fzds (table 1). We also detected expression of the Wnt antagonists Dkk1, Dkk4, sFRP4 and WIF1 mRNAs in the BM B progenitor cells (fig. 1 and table 1). Of these, sFRP4 mRNA was most readily detectable, suggesting the highest expression level. sFRP2 and sFRP3 mRNAs were variably detected (table 1), suggesting low expression levels.

**Table 1:** mRNA expression of Wnt ligands 1–19, Fzd receptors 1–10, Wnt antagonists sFRP1-4, WIF1, Dkk1 and Dkk4

	**BM B progenitor cells**	**BM stromal cells (BMS)**	**Human fetal brain**			**BM B progenitor cells**	**BM stromal cells (BMS)**	**Human fetal brain**

Wnt1	+/-	-	+		Fzd1	+/-	-	-
Wnt2	-	-	+		Fzd2	+	-	+
Wnt2B	+	+	+		Fzd3	+	+	+
Wnt3	-	-	+		Fzd4	+	+	+
Wnt3A	+/-	-	-		Fzd5	+	-	+
Wnt4	+/-	-	+		Fzd6	+	+	+
Wnt5A	+/-	+	+		Fzd7	+/-	-	+
Wnt5B	+	+	+		Fzd8	ND	-	ND
Wnt6	ND	-	ND		Fzd9	+	-	+
Wnt7A	-	-	+		Fzd10	-	-	-
Wnt7B	-	-	+		Dkk1	+	+	+
Wnt8A	+	-	+		Dkk4	+	-	-
Wnt8B	ND	+	ND		sFRP1	-	-	+
Wnt9A	+/-	-	+		sFRP2	+/-	+	+
Wnt9B	+/-	+	+		sFRP3	+/-	+	+
Wnt10A	+	-	-		sFRP4	+	-	+
Wnt10B	+/-	-	+		WIF1	+	ND	+
Wnt11	+/-	-	+				
Wnt16	+	-	+				

Genes expressed (+), not expressed (-), variably expressed between experiments(+/-), not determined (ND). N = 2. BM B progenitor cells: CD10^+^IgM^-^CD45^+ ^cells sorted by FACS and pooled from three different donors. Total RNA from human fetal brain was used as control.

RT-PCR performed on RNA from BM stromal cells showed expression of Wnt2B, Wnt5A, Wnt5B and Wnt8B. mRNA expression of Wnt9B was also demonstrated in these cells, although at a lower levels. Moreover, Fzd3, 4 and 6 mRNAs were detected in BM stromal cells, as well as expression of the Wnt antagonists Dkk1, sFRP2 and sFRP3 mRNAs (table 1).

### Regulated expression of Wnt receptors, β-catenin, plakoglobin, LEF-1 and TCF-4 mRNAs during human BM B lymphopoiesis

Identification of differential expression of Wnt signaling molecules during the B lymphopoiesis may reveal at which window in the process Wnt signaling is active. Thus, using quantitative real-time PCR, we examined the expression of a selection of Wnt receptors, β-catenin, plakoglobin and transcription factors in FACS sorted human BM B lineage cells representing different maturation levels; ELP cells (CD10^+^CD34^+^CD19^-^, also tested to be CD38^+^), pro-B cells (CD10^+^CD34^+^CD19^+^CD20^-^IgM^-^), large pre-B cells (CD10^+^CD34^-^CD19^+^CD20^dim^IgM^-^), small pre-B (CD10^+^CD34^-^CD19^+^CD20^-^IgM^-^) and immature B cells (CD10^+^CD34^-^CD19^+^CD20^+^IgM^+^). Due to limited number of cells, expression analysis in ELP cells was restricted to seven out of ten mRNAs.

**Figure 2:**
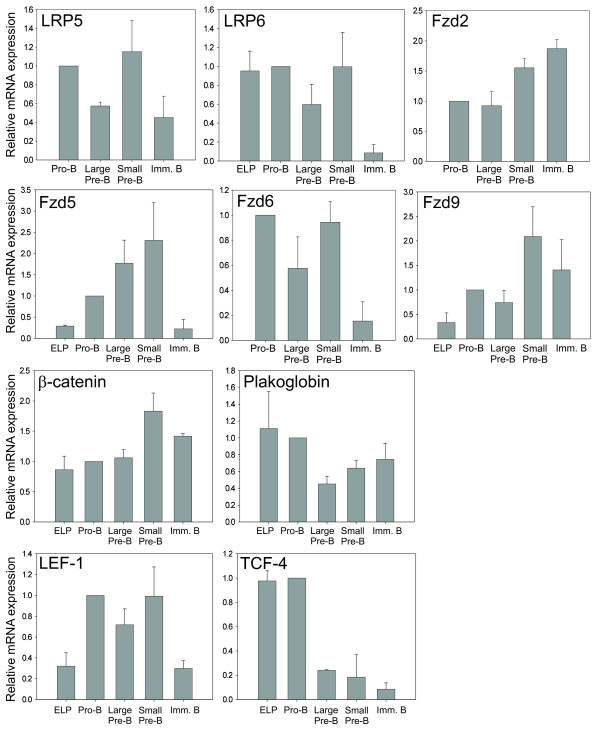
**Real-time PCR analysis of relative mRNA expression levels of Wnt pathway molecules in BM B progenitor sub-populations**. The sub-populations ELP, pro-B, large pre-B, small pre-B and immature B (imm.B) were isolated by FACS sorting. The relative mRNA expression levels of Wnt receptors and co-receptors, β-catenin, plakoglobin, LEF-1 and TCF-4 were quantified by real-time PCR analysis. Calculations of the expression levels were performed using the standard curve method and then normalized to the expression of PGK1 mRNA. mRNA levels in pro-B cells were used as calibrators. The bars represent the mean of 3–5 experiments ± SEM.

The results showed regulation of several of the important Wnt-signaling molecules, and different expression profiles were recognizable (fig. 2). mRNA levels for the plasma membrane receptors LRP5, LRP6, Fzd5 and Fzd6 dropped considerably as the cells develop from small pre-B cells into immature B cells. Furthermore, Fzd5 mRNA levels were strongly up-regulated as the cells commit to the B lineage (from ELP to pro-B), with a further up-regulation as the cells differentiate to pre-B cells. Fzd2 and Fzd9 mRNA levels, on the other hand, seemed to increase somewhat throughout the differentiation, with highest levels in small pre-B and immature B cells. In small pre-B cells, the mRNA levels of LRP5 and Fzd9 were about two-fold higher than in the large cycling pre-B cells. The expression levels of all receptors were low compared to the expression levels of e.g. LEF-1 and β-catenin, indicating relative low mRNA expression levels. Fzd3 and Fzd4 mRNAs were not detectable with the amount of RNA template used in these assays.

The mRNA expression of β-catenin and plakoglobin showed little variation as the cells differentiate. β-catenin mRNA was evenly expressed in ELP, pro-B, large pre-B and immature B, with a small increase (near two-fold) in small pre-B cells. Plakoglobin mRNA levels, in contrast, decreased 2-fold as the cells became large pre-B cells (fig. 2).

LEF-1 and TCF-4 mRNA expression is highly regulated during the early B lymphopoiesis, as shown previously by microarray analysis (Hystad ME *et al*, manuscript in preparation and [33]). Our results showed a strong up-regulation of LEF-1 mRNA as the cells commit to the B lineage, and the expression was kept continuously high until the cells become immature B cells, where the level was reduced to the same as in uncommitted progenitors. Here, low LEF-1 expression was further confirmed by the absence of LEF-1 protein in B lymphocytes from peripheral blood (results not shown). The relative TCF-4 mRNA levels, on the other hand, were high in both ELP and pro-B, and decreased (up to 5-fold) as the cells passed through Ig rearrangement (pre-B – immature B cells) (fig. 2). It should be noted that the LEF-1 mRNA expression was detected 5–8 cycles earlier than the TCF-4 mRNA expression, indicating that LEF-1 mRNA is much more abundant than TCF-4 mRNA.

### Wnt3A induces β-catenin stabilization and accumulation in BM B progenitor cells

**Figure 3:**
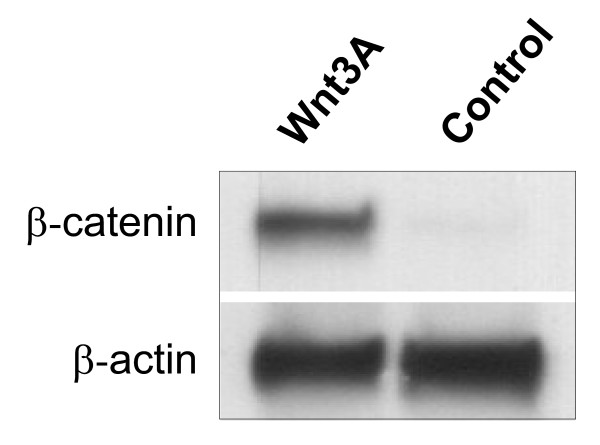
**Wnt3A induces β-catenin stabilization in BM B progenitor cells**. Western blot analysis of β-catenin levels in BM CD10^+ ^B lineage progenitor cells stimulated with Wnt3A (100 ng/ml) or vehicle (PBS with 0.1% detoxified BSA) for 3 hours. The blots were incubated with an Ab against β-catenin, followed by an Ab against β-actin to ascertain equal loading in the wells. The same results were found in cells from 4 out of 5 different donors, indicating some degree of donor variation in the response to Wnt3A.

Our data demonstrated that human BM B progenitor cells express a set of central players in the canonical Wnt signaling pathway, potentially allowing a Wnt signal to be conveyed. To further examine whether B progenitor cells could respond to treatment with Wnt proteins, we looked for the stabilization and subsequent accumulation of the vital signaling molecule β-catenin in CD10^+ ^B progenitor cells. When these cells were treated with Wnt3A, the amount of β-catenin increased substantially compared to the very low levels in untreated cells (fig. 3). Although there were some donor variations, the results showed that the B progenitor cells are able to receive and communicate a signal from the Wnt pathway.

### Wnt3A inhibits human *in vitro *B lymphopoiesis

Having identified expression of central molecules in the canonical Wnt pathway in BM B progenitor cells, we performed two variants of B lymphopoiesis assays to investigate whether Wnt signaling (using recombinant Wnt3A) had a functional effect on B lymphopoiesis *in vitro*. Both assays were based on coculture with the murine stromal cell line MS-5. In assay 1 hematopoietic progenitor cells (HPC) were tested for their capacity to develop into B lineage cells, whereas in assay 2 B progenitor cells were measured for survival and expansion. At the endpoint of the assays, each sample was subjected to quantitative flow cytometry and the total number of cells positive for the pan B cell marker CD19 was measured. In assay 2, analysis of the differentiation marker CD34 was included.

**Figure 4:**
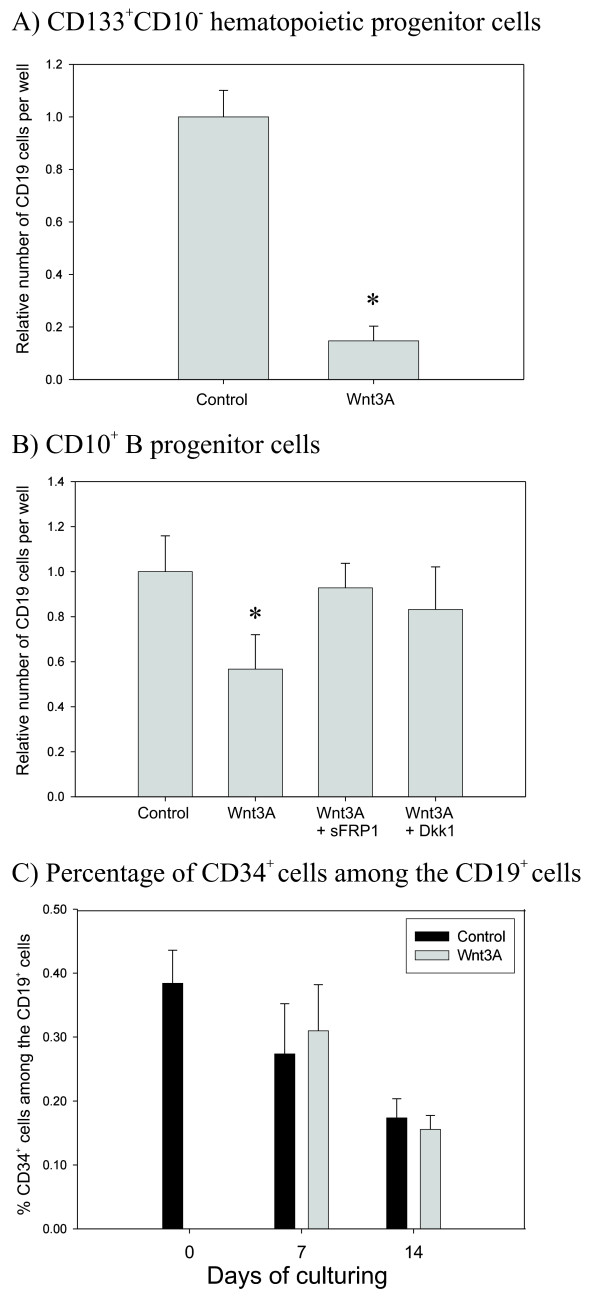
**Wnt3A inhibits *in vitro *B lymphopoiesis**. BM CD133^+^CD10^- ^HPC (A: assay 1) or CD10^+ ^B progenitor cells (B: assay 2) were cocultured with a confluent layer of the murine stromal cell line MS-5 for 3 or 2 weeks, respectively, while treated with Wnt3A (100 ng/ml), Wnt3A + sFRP1 (2 μg/ml), Wnt3A + Dkk1 (500 ng/ml) or medium only. The number of resulting CD19^+ ^B lineage cells in each sample was determined by quantitative flow cytometry. The percentage of CD34^+ ^cells among the CD19^+ ^cells were measured before and after culturing, with and without treatment with Wnt3A (C). The bars represent the mean of N experiments performed in duplicate, ± SEM. A) N = 6. B) Cells treated with control medium or Wnt3A: N = 11, Wnt3A + sFRP1: N = 3, Wnt3A + Dkk1: N = 2. C) day 0: N = 7, day 7: N = 3, Day 14: N = 8. *p ≤ 0.01, Wilcoxon Signed Ranks Test.

Initial analyses demonstrated that Wnt3A had an inhibitory effect when BM HPC (CD133^+^CD10^-^) were grown on stromal cells for 3 weeks at conditions that favored B lymphopoiesis (assay 1). The number of CD19^+ ^cells in the samples treated with Wnt3A was 5 times less than the number measured in the control samples (fig. 4A). The inhibited B lymphopoiesis could result from Wnt3A suppressing differentiation of the HSC pool found in the HPC population [34], an indirect effect mediated by the stromal cells [35], or, alternatively, Wnt3A could target more committed lymphoid progenitor cells. To examine the latter possibility in more detail, we tested whether Wnt3A acted on later stages of *in vitro *B lymphopoiesis. BM B progenitor cells (CD10^+^) were grown on stromal cells in the presence of Wnt3A or medium only for 2 weeks (assay 2). In accordance with the results from the assays using HPC, it was demonstrated on average near 50% reduction in the total number of CD19^+ ^cells in samples treated with Wnt3A compared with control (fig. 4B). When added every third day, both sFRP1 and Dkk1 were able to counteract the effect of Wnt3A almost completely, demonstrating a specific effect of Wnt3A on *in vitro *B lymphopoiesis (fig. 4B). Similar results were obtained using Wnt3A protein from another source; Wnt3A conditioned medium (table 2). Moreover, the effect was independent of the source of stromal cells as the use of primary human BM stromal cells (BMS) as supportive layer did not change the outcome of the experiment (table 2).

To check whether Wnt3A affected distinct early B subpopulations differently, the cells in assay 2 were additionally analyzed for expression of the CD34 differentiation marker to distinguish between pro-B and pre-B cells. The relative frequency of CD34^+ ^cells (pro-B) decreased from 38 % before culturing (day 0), to approximately 30 % and 15 % after one and two weeks of culturing, respectively. This decrease was independent of treatment with or without Wnt3A (fig. 4C). Furthermore, separation of the pre-B population into large cycling and small resting pre-B cells by surface expression of CD20 [33] revealed inhibitory effect of Wnt3A on all subpopulations (results not shown). Thus, we conclude that Wnt3A does not affect the relative proportions of different BM B subpopulations, but has a general inhibitory effect on pro-B, pre-B and immature B cells in a stroma coculture.

**Table 2:** Number of CD19 cells after two weeks of culturing BM CD10^+ ^cells on stromal cells

**Exp. No. (with MS5)**	**Control-CM**	**Wnt3A-CM**	Inhibition Index*
**1**	2182 ± 427	184 ± 91	0.08
**2**	9440 ± 1953	2652 ± 721	0.28
**3**	7292 ± 1928	2524 ± 475	0.35

**Exp. No. (with BMS)**	**Medium**	**rmWnt3A**	Inhibition Index*

**1**	1746 ± 300	920 ± 64	0.53

BM CD10^+ ^cells were cultured on a layer of the murine stromal cell line MS-5 in the presence of Wnt3A-conditioned medium (Wnt3A-CM) or control-conditioned medium (control-CM), or on a layer of human bone marrow stromal cells (BMS) in the presence of rmWnt3A or control medium. The numbers in the table represent the mean of duplicate wells ± SD. *Number of CD19^+ ^cells in wells containing Wnt3A divided by number of cells in wells containing control-medium.

### Wnt3A inhibits BM B progenitor cell division *in vitro*

**Figure 5:**
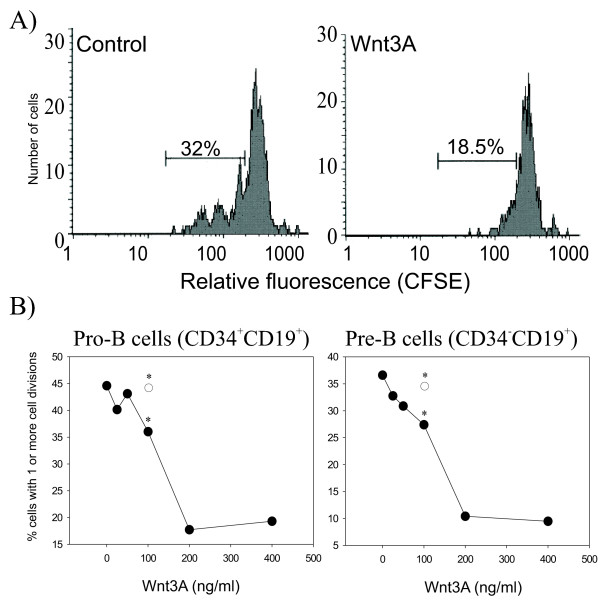
**Wnt3A inhibits the initial phase of stromal supported cell division of BM B progenitors**. Highly purified BM CD10^+^CFSE^mean ^cells were grown on a confluent layer of MS-5 and treated with Wnt3A (25–400 ng/ml) or medium only. After three days, the cells were analyzed on a FACScan flow cytometer for the number of cell divisions of CD19^+ ^cells. A) Tracking histograms of cell divisions of CFSE-labeled BM B progenitor cells in the presence or absence of Wnt3A (100 ng/ml) One representative experiment of six is shown. B) Dose dependent inhibition of cell division of CD34^+ ^pro-B cells and CD34^- ^pre-B cells by Wnt3A (closed circles). The inhibitory effect of Wnt3A was blocked by Wnt antagonist sFRP1 (2 μg/ml) (open circle). Data are shown as percentage of cells that had gone through one or more cell divisions, as determined by cell division tracking with CFSE. One representative experiment of two is shown, except for Wnt3A (100 ng/ml) and Wnt3A + FRP1 (2 μg/ml) where one representative experiment of six is shown (*p < 0.05, Wilcoxon Signed Ranks Test, n = 6).

The inhibitory effect of Wnt3A on *in vitro *B lymphopoiesis could be explained by increased apoptosis, an inhibitory effect on proliferation, or both. However, measurements of apoptosis in cells cultured without stromal cells for 1, 2 or 3 days showed no effect of Wnt3A (results not shown), suggesting an effect on proliferation only. To verify this, we used high-resolution cell division tracking to study the initial effects of Wnt3A on B progenitor cells grown on a stromal layer. Sorted CFSE labeled CD10^+ ^B progenitor cells were cocultured with MS-5 for 3 days in the presence of Wnt3A or medium only, and examined for the number of cell divisions by flow cytometry as well as the surface markers CD34 and CD19. The data clearly demonstrated that Wnt3A inhibited the initial divisions of B progenitor cells taking place in the coculture (fig. 5A). When gating for pro-B cells (CD34^+^CD19^+^) and pre-B cells (CD34^-^CD19^+^) separately, we found that Wnt3A inhibited proliferation of both these populations in a dose-dependent manner (fig. 5B). This effect was blocked by the Wnt antagonist sFRP1 (fig. 5B).

## Discussion

Several studies have identified the canonical Wnt pathway as a regulator of the homeostasis of human and murine HSC and hematopoietic progenitor cells [26,34,36]. Furthermore, knockout studies (LEF-1 and Fzd9) in mice have indicated a central role for Wnt signaling in B lymphopoiesis [24,27]. The Wnt pathway also seems to be involved in development of leukemia [28-30]. In the present work, we wanted to study in more detail the implications of canonical Wnt signaling in human BM B lymphopoiesis. Here, we describe that a set of Wnt ligands, Fzd receptors and Wnt antagonists is expressed in BM B progenitor cells, allowing a Wnt signal to be conveyed and modulated in these cells. We demonstrate regulated expression of several Wnt receptors, β-catenin and plakoglobin as well as the transcription factors LEF-1 and TCF-4 mRNAs during early differentiation steps in the B cell lineage, supporting the hypothesis that Wnt signaling is active in BM B lymphopoiesis. Furthermore, we show that canonical Wnt signaling, as measured by the accumulation of β-catenin levels, is induced in human BM B progenitor cells. Finally, we demonstrate that Wnt3A inhibits human stromal dependent B lymphopoiesis and that this effect is a consequence of decreased cell proliferation.

We show that CD10^+ ^human B progenitor cells express a set of Wnt ligand mRNAs (2B, 5B, 8A, 10A and 16), of which Wnt16 is of particular interest, since this gene is activated by the E2A-Pbx1 translocation in some cases of acute lymphocytic leukaemia (ALL) [28]. However, several pre-B leukemia cell lines studied [28] do not express Wnt16, suggesting a distinct role for this factor in early B lymphopoiesis that is turned off during leukemiagenesis, except in cases where Wnt16 is aberrantly activated by the E2A-Pbx1 fusion protein. Further, we demonstrate that primary BM stromal cells express mRNA of several Wnt ligands, including Wnt2B, Wnt5A, Wnt5B, Wnt8B and Wnt9B. This is partly in accordance with previous studies [24,26]. Taken together, these results show that both hematopoietic cells and the supporting stromal cells may produce Wnt ligands. Different Wnt ligands may have distinct effects during early B lymphopoiesis, which is a topic for future investigations.

So far, only scarce knowledge is available about both ligand specificity and tissue-restricted expression of the Fzd receptors. In our studies we found expression of a wide range of Fzd receptor mRNAs, including Fzd2, 3, 4, 5, 6 and 9, in BM B progenitor cells. Compared to the Wnt mRNAs, these are more readily detectable, indicating higher expression levels, which suggests that Wnt-signaling is important for B progenitor cells. Real-time PCR assays demonstrated differential expression of several Fzd receptor mRNAs, including Fzd5 and Fzd6, which are strongly down-regulated as the cells become immature B cells. Notably, LRP5 and LRP6 mRNAs showed a similar down-regulation. Furthermore, both Fzd5 and Fzd9 are up-regulated as the cells commit to the B cell lineage and go through differentiation. Interestingly, Fzd9^-/- ^mice show a depletion of developing B cells in the BM, particularly in the cycling pre-B population [27]. In contrast to this, our results show that the large cycling pre-B cells express lower levels of LRP5, LRP6, Fzd6, Fzd9, β-catenin and plakoglobin than the small resting pre-B cells. Although one should be cautious in trying to predict functional consequences from mRNA expression data, this trend suggests that Wnt signaling is not likely to be involved in a positive regulation of cycling of the large pre-B cells after Ig heavy chain rearrangement. And even though the absolute expression levels of the receptor mRNAs are low, these data suggest that during a narrow window of the development comprising pro- and pre-B cells, B progenitor cells might be target for Wnt signaling through these receptors.

To be able to convey a Wnt-signal, the cells have to express either of the two important molecules, β-catenin or plakoglobin. Our results show that levels of β-catenin mRNA change little during the differentiation. Although it has been demonstrated that levels of cytoplasmic β-catenin protein may vary throughout the development of thymocytes [37], these variations may not necessarily be reflected by the mRNA levels. In fact, as β-catenin is needed both for signaling purposes as well as for adhesion purposes, the mRNA levels may have to be kept relatively stable. Plakoglobin mRNA, on the other hand, decreases after the pro-B differentiation level. This corresponds to the observations made in developing murine thymocytes [37], where plakoglobin is down-regulated at the level of immature single positive thymocytes, suggesting that plakoglobin may play a central, but hitherto unexplored role in conveying a Wnt signal during lymphopoiesis. In fact, the lack of effect of knocking down β-catenin in early hematopoiesis, including B and T lymphopoiesis [38], prompted the authors to suggest that plakoglobin may stand-in for β-catenin in this respect.

The LEF-1/TCFs are directly activated by canonical Wnt signaling, and LEF-1 knockout mice show defects in pro-B cell proliferation and survival [24]. However, it cannot yet be ruled out that this effect might be a result of abolishment of the repressive functions or other non-Wnt related activities of LEF-1 [15]. Here, we have verified microarray data showing regulation of LEF-1 and TCF-4 during B lymphopoiesis (Hystad ME *et al*, manuscript in preparation and [33]). Interestingly, it has been reported that LEF-1 is a target gene for the B lymphopoiesis key transcription factor Pax-5 [39]. Moreover, LEF-1 interacts with Pax-5 and c-Myb to activate the Rag-2 promoter [40], but the accurate role of LEF-1 in B lymphopoiesis is still elusive. In contrast to LEF-1, we found TCF-4 mRNA levels to be high in ELP and pro-B cells, and lower in the more mature pre-B and immature B populations. Although expressed at lower levels, one could speculate that TCF-4 steps in for LEF-1 in the earliest lymphoid progenitors before LEF-1 is properly switched on, potentially in conveying a Wnt signal or, alternatively, in acting as a transcription repressor of B lineage genes before commitment. These are topics for further studies.

Wnt antagonists play important roles in preventing or fine tuning the Wnt signal [16]. Our data show expression of the Wnt antagonists Dkk1, Dkk4, sFRP4 and WIF1 mRNAs in B progenitor cells. Dkk1, sFRP2 and sFRP3 were expressed in bone marrow stromal cells. Of these factors, Dkk1 in particular is known to be involved in a feedback loop to adjust or shut down canonical Wnt signaling [41]. It is likely that these factors are important in adjusting the incoming Wnt signals in the bone marrow microenvironment, where several cell types are able to express a wide range of ligands and Wnt receptors.

The inhibitory effect of Wnt3A on the generation and cell division of B progenitor cells *in vitro*, both with regard to pro- and pre-B cells, is in contrast to several reports on the functional effects of canonical Wnt signaling in mice. Both in murine HSC [34], developing thymocytes [25] and a wide range of cancer cells [31,42], elevated levels of β-catenin lead to increased cell proliferation. Furthermore, in fetal murine pro-B cell [24], Wnt3A conditioned medium leads to increased BrdU incorporation. Our divergent results may be due to different species, microenvironments and/or cell context. For instance, murine and human B lymphopoiesis require to a certain extent differing factor dependency [32]. However, by culturing murine BM B progenitor cells, we have not been able to demonstrate increased cell proliferation in the presence of Wnt3A (results not shown). Thus, we suspect the Wnt response to be different in fetal and adult B progenitor cells, potentially affected by the cellular microenvironment and/or context. Indeed, the fetal pro-B cells are exposed to the microenvironment of the liver and this is very different from that of the BM. For instance several regulators of the Wnt pathway are more highly expressed in fetal liver stroma than in BM stroma [43], which suggest that Wnt signaling might be regulated in a different manner and have a different role in the fetal liver than in the BM. Another important aspect that has to be taken into consideration, is that different Wnt ligands, although able to activate canonical Wnt signaling, indeed show distinct activities [44]. In addition there may also be species and location differences. However, as mentioned above, Cobas *et al *have demonstrated a lack of an essential role for β-catenin in BM hematopoiesis, including proliferation of B lymphocytes [38]. Thus, in contrast to findings in the fetal liver, our results may very well represent a physiological situation in the adult organism, where Wnt signaling via β-catenin is not essential for B lymphocytes, but may be used to fine tune the delicate balance between proliferation, differentiation and apoptosis taking place during early BM B lymphopoiesis.

In support of our data on an inhibitory effect of Wnt3A on cell division, it has been reported that canonical Wnt signaling hampers fibroblast cell proliferation through cell cycle blocks, potentially mediated via p53 [45]. Moreover, Wnt signaling inhibits proliferation and regulates cell-cycle arrest at distinct stages of development in Drosophila wing development [46]. Thus, it is likely that the cellular context, in some cases represented by the ability of a central regulatory molecule like p53 to respond, will affect how the cells react to vital stimuli like Wnt. It has been speculated that aberrant p53 is necessary to convey the strong tumor promoting effect of abnormal Wnt signaling seen in colon cancer [47,48]. It is also interesting that Wnt5A has been found to inhibit B cell proliferation and can function as a tumor suppressor in hematopoietic tissue, albeit via the non-canonical Wnt/Ca^2+ ^pathway [49].

We show expression of Wnt2B, 5B, 8A, 10A and 16 in BM CD10^+ ^cells and of Wnt2B, Wnt5A, Wnt5B, Wnt8B and Wnt9B mRNAs in human primary BMS cells. Further we demonstrate that Wnt3A acts directly on B progenitor cells by increasing the levels of β-catenin, suggesting that the microenvironment may use Wnt signaling to regulate the fate of developing B lymphocytes. Yet, we cannot exclude that the functional effect of Wnt3A on *in vitro *B lymphopoiesis is indirect and mediated via the stromal cells, as observed for *in vitro *hematopoiesis [35]. The BM microenvironment is composed of a heterogeneous population of cells including fibroblasts, adipocytes, endothelial cells and osteoblasts, all derived from a common mesenchymal precursor [50]. In particular, the role of Wnt signaling in adipogenesis may be relevant here, as it has been demonstrated that Wnt10B [51,52] inhibits adipogenesis, and there seems to be a positive correlation between adipogenesis and hematopoiesis [52]. This emphasizes the complexity of the interactions in the B lymphopoiesis maturation niche and opens for the possibility that B progenitor cells may manipulate the stromal support via these Wnt factors. However, it is not uncommon in developmental niches that morphogenic signals have the potential to act on several cells in the microenvironment. Therefore, it has been suggested that Wnt signaling might influence the HSCs both directly and indirectly by maintenance of the cellular elements of the stem cell niche [21]. In line with this theory, several studies have demonstrated expression of multiple Wnt mRNAs in thymocytes and the thymic microenvironment. It is likely that particular Wnts serve distinct roles, thus, cell specific effects may be achieved by "playing the Wnt repertoire" as well as through combinations with other signaling events.

## Conclusion

In this study, we have demonstrated mRNA expression of several Wnt ligands, Fzd receptors and Wnt antagonists in human BM B progenitor cells and regulated expression of Fzd receptors and co-receptors, β-catenin, plakoglobin, LEF-1 and TCF-4 mRNA in these cells during differentiation. Furthermore, we find that Wnt3A induced an accumulation of β-catenin in the BM B progenitor cells and inhibition of *in vitro *B lymphopoiesis. These results suggest the Wnt/β-catenin pathway as a negative regulator of human stromal dependent B lymphopoiesis. This is in contrast to observations on Wnt effects in fetal murine pro-B cells, and may represent a distinction between the fetal liver and adult BM microenvironments.

## Methods

### Reagents and antibodies for FACS and western blot analysis

Recombinant murine (rm) Wnt3A, recombinant human (rh) secreted frizzled related protein 1 (sFRP1), rh Dickkopf 1 (Dkk1), rh interleukin (IL)-7, rh IL-3 and rh Flt3 ligand (FL) were purchased from R&D Systems (Great Britain). The following monoclonal antibodies (mAbs) were used for flow cytometry: anti-CD34 PE, anti-CD10 APC, anti-CD10 PE-Cy7 and anti-CD20 APC from Becton Dickinson, Biosciences Pharmingen (San Jose, CA, USA), anti-CD19 PE-Cy5 and anti-CD34 PE-Cy5 from Immunotech (Marseille, France) and anti-CD19 PE, anti-CD45 PE and anti-IgM Fitc from Dako Cytomation (Denmark). Irrelevant isotype matched Abs were used as controls. The following Abs were used in western blot analysis: anti-β-catenin (Mouse IgG1, BD Transduction Laboratories), anti-β-actin (Goat polyclonal IgG, Santa Cruz Biotechnology), rabbit anti-mouse IgG1-HRP and rabbit anti-goat IgG-HRP (Dako cytomation, Denmark).

### Primary cells and cell lines

BM aspirates were from the iliac crest of normal adult volunteers (approved by the Regional Ethical Committee). Mononuclear cells (MNC) were separated by Ficoll-Hypaque density gradient centrifugation (Lymphoprep, Nycomed, Norway). CD10^+ ^B progenitor cells (ELP, pro-B and pre-B cells) were isolated from BM MNC using Dynabeads^®^M-450 Epoxy (Dynal, Oslo, Norway) directly coated with anti-CD10 mAb (clone RFAL-3, Sigma-Aldrich, UK) followed by detachment using CD4/CD8 DETACHaBEAD (Dynal, Norway) according to the producer 's protocol. The CD10^+ ^cells were of 90–95% purity, they were CD45^+ ^and contained 4–7% IgM^+ ^cells (immature B cells). CD34^+ ^and CD19^+ ^cells were isolated in a similar manner from MNC using Dynabeads^®^M-450 conjugated with anti-CD34 or anti-CD19 mAb, respectively, and CD34 or CD19 DETACHaBEAD (Dynal, Norway), respectively. CD133^+^CD10^- ^cells (HPC) were isolated from the CD10^- ^fraction of BM MNC (see above) using the MACS system (Magnetic cell sorting of human cells) and a CD133 Cell Isolation Kit (Miltenyi Biotec, Germany). Briefly, the mononuclear cells were magnetically labeled with CD133 MicroBeads and separated on a column, which was placed in the magnetic field of a MACS Separator. The magnetically labeled CD133^+ ^cells were retained in the column while the unlabeled CD133^- ^cells passed through. After removal of the column from the magnetic field, the magnetically retained CD133^+ ^cells were eluted as the positively selected cell fraction. The CD133^+ ^cells were typically of 97–98% purity. In monoculture, the cells were kept in X-VIVO 15™ (BioWhittaker, Walkersville, USA) with 0.1% detoxified BSA.

The murine stromal cell line MS-5 [53] was cultured in α-MEM with 10% FCS and 100 μg/ml of penicillin and streptomycin (PAA Laboratories, Pasching, Austria) and was passaged twice a week. Cultures of human BM stromal (BMS) cells were established as previously described [54]. Briefly, total BM MNC cells depleted of CD34^+ ^cells were seeded into 75-cm^2 ^tissue culture flasks in RPMI-1640 with 10% FCS, penicillin and streptomycin. Non-adherent cells were washed off after 2 hours at 37°C, and the adherent cells were cultured in EX-CELL 610 (JRH Biosciences, USA) with 10% FCS, penicillin and streptomycin. The BMS cells were passaged twice before they were used for experiments.

The human ALL cell lines Reh (no ACC 22, DSMZ) and Nalm-6 (no ACC 128, DSMZ) (Hurwitz et al, 1979) were kept in X-VIVO 15™ supplemented with 100 μg/ml of penicillin and streptomycin.

### FACS analysis and cell sorting

Cells were stained with anti-CD19 PE Ab for 30 min at 4°C before analysis on FACSCalibur flow cytometer (argon-ion laser tuned at 488 nm; Becton Dickinson). Quantitative analysis of CD19^+ ^cells in cocultures was performed using Flow Cytometry Absolute Count Standard, from Bangs Laboratories Inc., (Fishers, IN 46038 USA). Data acquisition and analysis were performed using CELLQuest software (Becton Dickinson).

Highly purified (98–99%) B progenitor cells for RT-PCR analysis of Wnt, Fzd and Wnt antagonist mRNA expression were obtained by sorting of BM CD10^+^CD45^+^IgM^- ^B progenitor cells using a FACSDiVa flow cytometer (Becton Dickinson, USA) after staining of BM CD34^+ ^and CD19^+ ^isolated cells with anti-CD45 PE, anti-CD10 APC and anti-IgM FITC Abs. Highly purified BM cell populations for real-time PCR were obtained by staining BM CD34^+ ^and CD19^+ ^cells with anti-CD10 PE-Cy7, anti-CD34 PE, anti-CD19 PE-Cy5, anti-CD22 APC and anti-IgM Fitc Abs and the following subpopulations were sorted using a FACSDiVa flow cytometer: ELP (CD10^+^CD34^+^CD19^-^IgM^-^), pro-B (CD10^+^CD34^+^CD19^+^CD20^-^IgM^-^), large pre-B (CD10^+^CD34^-^CD19^+^CD20^dim^IgM^-^), small pre-B (CD10^+^CD34^-^CD19^+^CD20^-^IgM^-^) and immature B (CD10^+^CD34^-^CD19^+^CD20^high^IgM^+^). Separation of large and small pre-B cells was based on both CD20 expression and size (forward scatter, FSC).

### PCR analysis

Total RNA from freshly isolated and sorted BM CD45^+^CD10^+^IgM^- ^cells was isolated using Absolutely RNA™ RT-PCR Mini-prep kit (Stratagene Europe, Amsterdam, Netherland) according to the manufacturer's instructions. RNA from human fetal brain was purchased from BioChain Institute, Inc., USA. cDNA was synthesized from 1 μg total RNA primed with random hexamers in a 50 μl reaction using TaqMan Reverse Transcription Reagents (Applied Biosystems, Foster City, CA, USA). Control reactions lacking reverse transcriptase were always included. RT-PCR of 20 ng of total RNA was performed with a titanium polymerase (BD Biosciences, USA) in a 25 μl reaction for 37 cycles at 95°C for 30 seconds, 60°C for 30 seconds, and 68°C for 30 seconds. The primer sequences used to identify Wnt, Fzd and Wnt antagonist gene expression are listed in Table 3. The primer sequences was partly designed specifically for this work and partly copied from previous expression analyses [55]. For all mRNAs expressed, the amplified products have been sequenced and confirmed to represent the correct target gene.

**Table 3:** Primer sequences used for mRNA expression analyses of Wnt ligands, Fzd receptors and Wnt antagonists

*Primer*	*Sequence*	*Amplicon (bp)*
Wnt1	F-5' TAG CCT CCT CCA CGA ACC TG-3'	239
	F-5' CAG CCT CGG TTG ACG ATC TTG-3'	
Wnt2	F-5' TGG TGG TAC ATG AGA GCT ACA GGT G-3'	297
	R-5' CCC TGG TGA TGG CAA ATA CAA C-3'	
Wnt2B	F-5' TCA TGC TCA GAA GTA GCC GAG A -3'	328
	R-5' TGG CAC TTA CAC TCC AGC TTC A -3'	
Wnt3	F-5' CTG GCT ACC CAA TTT GGT GGT-3'	225
	R-5' CAT CTA TGG TGG TGC AGT TCC A-3'	
Wnt3A	F-5' AAG CAG GCT CTG GGC AGC TA-3'	234
	R-5' GAC GGT GGT GCA GTT CCA-3'	
Wnt4	F-5' GAG GAG ACG TGC GAG AAA CTC AA-3'	346
	R-5' ATC CTG ACC ACT GGA AGC CCT GT-3'	
Wnt5A	F-5' ATC CTG ACC ACT GGA AGC CCT GT-3'	358
	R-5' GGC TCA TGG CGT TCA CCA C-3'	
Wnt5B	F-5' CAG CTT CTG ACA GAC GCC AAC T-3'	279
	R-5' GCC TAT CTG CAT GAC TCT CCC A-3'	
Wnt6	F-5' GCT CCA GCC ACA GCA AGG-3'	378
	R-5' CAG CCT GCC CGC CTC GTT-3'	
Wnt7A	F-5' CCT GGG CCA CCT CTT TCT CAG-3'	573
	R-5' TCC AGC TTC ATG TTC TCC TCC AG-3'	
Wnt7B	F-5' TTT CTC TGC TTT GGC GTC CT-3'	391
	R-5' TGG TTG TAG TAG CCC TGC TTC TC-3'	
Wnt8A	F-5' TCC CAA GGC CTA TCT GAC CTA C-3'	400
	R-5' CCG GCC CTG TTG TTG TGA-3'	
Wnt8B	F-5' GCC CAG AGT GGT ATT GAA GAA TG-3'	266
	R-5' TTG TCA CTG CAG CCT CCC-3'	
Wnt9A	F-5' AAG TAC AGC AGC AAG TTC GTC AAG G-3'	538
	R-5' GCA CTC CAC ATA GCA GCA CCA AC-3'	
Wnt9B	F-5' AGT TTC AGT TCC GGC ATG AGC-3'	340
	R-5' TTC ACA GCC TTG ATG CCC A-3'	
Wnt10A	F-5' ACA CAG TGT GCC TAA CAT TGC C-3'	296
	R-5' AGG CCT TCA GTT TGC CCA G -3'	
Wnt10B	F-5' CCT CGC GGG TCT CCT GTT C-3'	563
	R-5' GGT TAC AGC CAC CCC ATT CC-3'	
Wnt11	F-5' ACA ACC TCA GCT ACG GGC TCC T-3'	394
	R-5' CCC ACC TTC TCA TTC TTC ATG C-3'	
Wnt16	F-5' CTG TGC AAG AGG AAA CCG TAC CTG-3'	520
	R-5' CAG CAC AGG AGC CGG AAA CT-3'	
Fzd1	F-5' CTC TAC TTC TTC AGC ATG GCC A-3'	230
	R-5' TCC ACG TTG TTA AGC CCC A-3'	
Fzd2	F-5' CCA TCC TAT CTC AGC TAC AAG TTT CT-3'	306
	R-5' GCA GCC CTC CTT CTT GGT-3'	
Fzd3	F-5' TCC CCT CTG CCT GTA TGT GGT AGT-3'	622
	R-5' GCT GCT CAC TTT GCT GTG GA-3'	
Fzd4	F-5' CTC GGC TAC AAC GTG ACC AAG AT-3'	605
	R-5' AAT ATG ATG GGG CGC TCA GGG TA-3'	
Fzd5	F-5' GTG CCC ATT CTG AAG GAG TCA C-3'	197
	R-5' TCC ATG TCG ATG AGG AAG GTG-3'	
Fzd6	F-5' ACT CTT GCC ACT GTG CCT TTG-3'	300
	R-5' GTC GAG CTT TTG CTT TTG CCT-3'	
Fzd7	F-5' CAA GAC CGA GAA GCT GGA GAA G-3'	248
	R-5' TGC CGA CGA TCA TGG TCA T-3'	
Fzd8	F-5' GGA CTA CAA CCG CAC CGA CCT-3'	407
	R-5' ACC ACA GGC CGA TCC AGA AGA C-3'	
Fzd9	F-5' TCA AGG TCA GGC AAG TGA GCA-3'	210
	R-5' AGC TTC CAG AGG AAC GCA ACA-3'	
Fzd10	F-5' CAG GTG TGC AGC CGT AGG TTA A-3'	212
	R-5' AAG CAC CAC ATC TTA GCT CCG G-3'	
WIF1	F-5' ACG GAC CTC ACT GTG AGA AAG C-3'	200
	R-5' GCT GAT TTC ACA CTG CTC TCC C-3'	
sFRP1	F-5' GGT CAT GCA GTT CTT CGG CT-3'	206
	R-5' TCC TCA GTG CAA ACT CGC TG-3'	
sFRP2	F-5' ACC GAG GAA GCT CCA AAG GTA T -3'	259
	R-5' TCA TCT CCT CAC AGG TGC ACT G -3'	
sFRP3	F-5' CTC ATC AAG TAC CGC CAC TCG TG-3'	210
	R-5' CCG GAA ATA GGT CTT CTG TGT AGC TC-3'	
sFRP4	F-5' GCA CAT GCC CTG GAA CAT CAC-3'	243
	R-5' ATC TTC ATG AGG GGC TCG CAG T-3'	
Dkk1	F-5' ACC ATT GAC AAC TAC CAG CCG T -3'	235
	R-5' TGG TTT CCT CAA TTT CTC CTC G -3'	
Dkk4	F-5' CGT TCT GTG CTA CAT GTC GTG G-3'	241
	R-5' TCT TGT CCC TTC CTG CCT TGT-3'	

### Real-time PCR

Total RNAs from 5–20 000 freshly isolated and sorted BM B progenitor cells (ELP, pro-B, large pre-B, small pre-B and immature B cells) were purified using Absolutely RNA™ RT-PCR Micro-prep kit (Stratagene Europe, Amsterdam, Netherland) according to the manufacturer's instructions. cDNAs were synthesized from total RNA primed with random hexamers using TaqMan Reverse Transcription Reagents (Applied Biosystems, Foster City, CA, USA). LEF-1 and TCF-4 (gene name TCF-7L2) mRNA expression was analyzed by real-time quantitative RT-PCR using Taqman technology according to the manufacturers procedure (Applied Biosystems). Predeveloped assay reagents including primers and probes for LRP5 (Hs00182031_m1), LRP6 (Hs00233935_m1), Fzd2 (Hs00361432_s1), Fzd5 (Hs00361869_g1), Fzd6 (Hs00171574_m1), Fzd9 (Hs00268954_s1), β-catenin (CTNNB1, Hs00170025_m1), plakoglobin (JUP, Hs00158408_m1), LEF-1 (Hs00212390_m1) and TCF-4 (Hs00181036_m1) mRNAs as well as the endogenous control phosphoglycerate kinase 1 (PGK1) (Hs99999906_m1) were supplied by Applied Biosystems and the PCR reactions were performed according to the manufacturer's instructions using Taqman Universal PCR Master Mix. Each measurement was performed in duplicate and the expression level for each gene was calculated using the standard curve method for relative quantitation of gene expression as described by the manufacturer (ABI Prism 7700 Sequence Detection System, User Bulletin 2, PE Applied Biosystems, Foster City, CA). Total RNA from the ALL cell lines Reh and Nalm6 as well as total RNA from human fetal brain were used for standard curves. Expression values for PGK1 mRNA, initially determined to be a suitable endogenous control for BM populations, were used for normalization of the expression levels. The expression level of the different genes in pro-B cells was used as a calibrator, and the expression of the other populations were calculated relative to the expression in pro-B cells.

### Western blot analysis

The cells were treated with Wnt3A or vehicle only (PBS with 0.1% detoxified BSA) for 3 hours and total cell lysates were analyzed by Western blot using 10% SDS polyacrylamide gels from Pierce (Rockford, USA) as described earlier [56]. The filters were pretreated with PBS containing 0.1% Tween-20 (PBS-T) and 5% dry milk, incubated overnight with anti-β-catenin Ab or 1 hour with anti-β-actin Ab and then washed 2 × 15 min in PBS-T. The filters were then incubated with the secondary Ab rabbit anti-mouse IgG1-HRP Ab or rabbit anti-goat IgG-HRP Ab, respectively, for 60 minutes at room temperature and washed 2 × 15 min in PBS-T before the proteins were visualized using ECL^+ ^Western Blotting Detection Reagents from Amersham Biosciences (Piscataway, NJ, USA).

### Hematopoietic cell-stromal cell coculture

Assay 1: HPC (CD133^+^CD10^-^) were cultured in 24 well tissue plates (2000 cells/well) pre-seeded with MS-5 (2.5 × 10^4 ^cells/well). Assay 2: B progenitor cells (CD10^+^) were cultured in 96 well tissue plates (8000 cells/well) pre-seeded with MS-5 (1 × 10^4 ^cells/well). Both sets of cocultures were in α-MEM containing 1% FCS, 100 μg/ml of penicillin and streptomycin, and supplemented with cytokines (for HPC: SCF, 25 ug/ml and G-CSF, 2,5 ug/ml, for B progenitor cells: IL-7, 50 ng/ml, IL-3, 20 ng/ml and FL, 50 ng/ml). In one additional experiment, the wells were pre-seeded with BMS (1 × 10^4 ^cells/well) in EX-CELL 610 with 1% FCS, 100 μg/ml of penicillin and streptomycin and cytokines (IL-7, 50 ng/ml, IL-3, 20 ng/ml and FL, 50 ng/ml). Where indicated, Wnt3A (10–100 ng/ml), Dkk1 (500 ng/ml) or sFRP1 (2 μg/ml) were added to the cultures. 50% of the medium was replaced weekly. After 3 (HPC) or 2 (B progenitor cells) weeks of culturing, single wells were harvested by trypsination and the B progenitor cells were immunophenotyped using the pan B cell marker CD19 as well as the CD34 differentiation marker and subjected to quantitative analyses (see above). Wnt3A conditioned medium and control medium were collected from L-Wnt3A cells and control nontransfected L-cells, respectively (purchased from American Type Culture Collection (ATCC), Manassas, USA), according to the manufacurer's procedure.

### High-resolution cell division tracking

BM CD34^+ ^and CD19^+ ^cells were labeled with 5- and 6-carboxyfluorescein diacetate succinimidyl ester (CFSE; Molecular Probes, Eugene, OR, USA) as described earlier [57]. To allow unbound dye to diffuse from cells, labeled cells were seeded on a confluent layer of MS-5 and incubated for 18–24 hours at 37°C in α-MEM with 1% FCS. Subsequently, the cells were stained with CD10 APC mAb and CD10^+^CFSE^mean ^cells were sorted on a BD FACSDiVa flow cytometer (Becton Dickinson). Sorted cells (1.5–2 × 10^4^/well) were cultured in 48 well tissue plates pre-seeded with MS-5 (2 × 10^4 ^cells/well) supplemented with IL-7 (50 ng/ml) and FL (50 ng/ml) and treated with Wnt3A (25–400 ng/ml), Wnt3A + sFRP1 (2 μg/ml) or medium only. IL-3 was left out of these cultures, because earlier experiments showed that IL-7 and FL were sufficient to support survival and proliferation of the B progenitor cells (data not shown). After three days the cells were harvested by trypsination and analyzed on a FACSCalibur flow cytometer for the number of cell divisions as well as expression of the cell surface markers CD34 and CD19.

### Statistical analysis

The statistical significance of differences between groups was determined using the paired two-tailed Wilcoxon's nonparametric test, by applying SPSS 11.5 software.

## Abbreviations

Wnt, Wingless-type MMTV integration site family

BM, bone marrow

Fzd, Frizzled

HSC, hematopoietic stem cell

HPC, hematopoietic progenitor cell

CLP, common lymphoid progenitor

ELP, early lymphoid progenitor

IL-7, interleukin 7

IL-3, interleukin 3

FL, Flt3 ligand, Fms-related tyrosine kinase 3 ligand

SDF-1, stromal cell-derived factor 1

Dkk, Dickkopf

sFRP, soluble Fzd related protein

GSK3β, glycogen synthase kinase-3β

LRP, low density lipoprotein receptor-related protein

LEF-1, lymphoid enhancer-binding factor 1

TCF-4, transcription factor 4

WIF1, Wnt inhibitory factor 1

CFSE, carboxyfluorescein diacetate succinimidyl ester

ALL, acute lymphocytic leukaemia

CLL, chronic lymphocytic leukaemia

BSAP, B-cell lineage specific activator protein

Pax-5, paired box gene 5

Rag-2, Recombination-Activating Gene-2

Rm, recombinant murine

Rh, recombinant human

mAb, monoclonal antibody

MNC, mononuclear cells

BMS, bone marrow stroma

BSA, bovine serum albumin

PGK1, phosphoglycerate kinase 1

## Authors' contributions

GD designed and conducted experiments, oversaw research, and wrote paper. ET designed and conducted experiments, oversaw research, and wrote paper. MKN designed and conducted experiments, oversaw research. HS designed and conducted experiments. SF designed experiments, oversaw research, and wrote paper. ER designed experiments, oversaw research, and wrote paper.
